# Feasibility and effectiveness of a single-catheter approach for adrenal vein sampling in patients with primary aldosteronism

**DOI:** 10.1186/s12902-021-00685-x

**Published:** 2021-01-30

**Authors:** Jindong Wan, Fei Ran, Siwei Xia, Jixin Hou, Dan Wang, Sen Liu, Yi Yang, Peng Zhou, Peijian Wang

**Affiliations:** 1 Department of Cardiology, The First Affiliated Hospital, Chengdu Medical College, Chengdu, 610500 Sichuan China; 2 Key Laboratory of Aging and Vascular Homeostasis of Sichuan Higher Education Institutes, The First Affiliated Hospital, Chengdu Medical College, Chengdu, 610500 Sichuan China

**Keywords:** Adrenal vein sampling, Primary aldosteronism, Single catheter, Adrenal adenomas, Hypertension

## Abstract

**Background:**

Adrenal vein sampling (AVS) is the preferred method for subtyping patients with primary aldosteronism, while the procedure is technically challenging. This study evaluated the feasibility and effectiveness of a single-catheter approach for AVS.

**Methods:**

A retrospective analysis of 106 consecutive patients who underwent AVS was performed to determine the procedural success and complication rates. Bilateral AVS procedures were performed using a single 5-Fr Tiger catheter with repeated manual reshaping.

**Results:**

We successfully advanced the catheter into the bilateral adrenal veins of all patients and reached a 90.6% procedural success rate of AVS. The procedural period was 33.0 ± 8.2 min, the fluoroscopy period was 5.8 ± 1.7 min, and the diagnostic contrast used was 17.3 ± 5.5 ml. Only one patient (0.9%) had a hematoma at the femoral puncture site. No other complications were observed. The operation period gradually shortened as the cumulative number of operations increased. The number of procedures required to overcome the learning curve was about 33 cases.

**Conclusions:**

The single-catheter approach is feasible and effective for AVS. Moreover, this approach required a relatively short learning curve for an inexperienced trainee.

## Background

Approximately 44.7% of Chinese adults aged 35–75 years have hypertension, and it was estimated that 244.5 million Chinese adults experienced hypertension [1, 2]. Primary aldosteronism (PA) is the most common cause of secondary hypertension with a prevalence of at least 4% in patients with newly diagnosed hypertension in Chinese population [3]. PA is associated with increased risk of cardiovascular disease compared with patients with primary hypertension [4]. Fortunately, nearly half of patients with PA that is due to an unilateral aldosterone-producing adenoma can be cured by adrenalectomy [5]. Aldosterone-producing adenoma and bilateral idiopathic adrenocortical hyperplasia are the two most common types of PA, while patients with the latter are unsuitable for adrenalectomy but need lifelong treatment with mineralocorticoid receptor antagonists. Therefore, appropriately selecting patients for unilateral adrenalectomy is critical in the differential diagnosis and subtyping of patients with PA. The current clinical practice guidelines recommend adrenal vein sampling (AVS) as a preferred method to differentiate unilateral from bilateral PA and to select patients with PA for unilateral adrenalectomy [6–8].

Although AVS is accepted as the gold standard test for subtyping PA, the procedure is underused especially in underdeveloped areas partially because the AVS procedure is technically challenging and relatively expensive [9]. Due to the anatomical complexity and variations, AVS fails frequently even in experienced hands [10]. In addition, the AVS procedure can have various complications including hematoma at the puncture site, venous dissection and thrombosis, and adrenal hemorrhage and insufficiency. Previous studies reported variable success rates of the AVS procedure ranging from 40 to 94% [10–12]. Catheterization into the right adrenal vein is particularly difficult as it is a small and short vessel which directly drains into the inferior vena cava (IVC). Because of the distinct anatomy of the left and right adrenal veins, two different catheters are usually used in the AVS procedure, which may increase the cost of this invasive test.

As mentioned, AVS is an optimal test for differential diagnosis and subtyping of patients with PA, but it is an invasive, relatively expensive procedure with technical challenges and varied success rates. The present study aimed to evaluate the feasibility and effectiveness of a single-catheter approach for AVS in patients with PA.

## Methods

### Patients

This retrospective study was approved by the Institutional Review Board of the First Affiliated Hospital of Chengdu Medical College. All patients had given their written consent for the AVS procedure and participation in the study. We enrolled 106 consecutive patients who were diagnosed PA and underwent the AVS procedure from October 2018 to April 2020. We only used the single-catheter technique for AVS during this period of time in our center. The inclusion criteria included diagnosis of PA by clinical and laboratory examinations including saline-loading test and completion of adrenal computed tomography scan. Patients with severe hepatic and renal disorders were excluded.

### AVS procedure

**Fig. 1:**
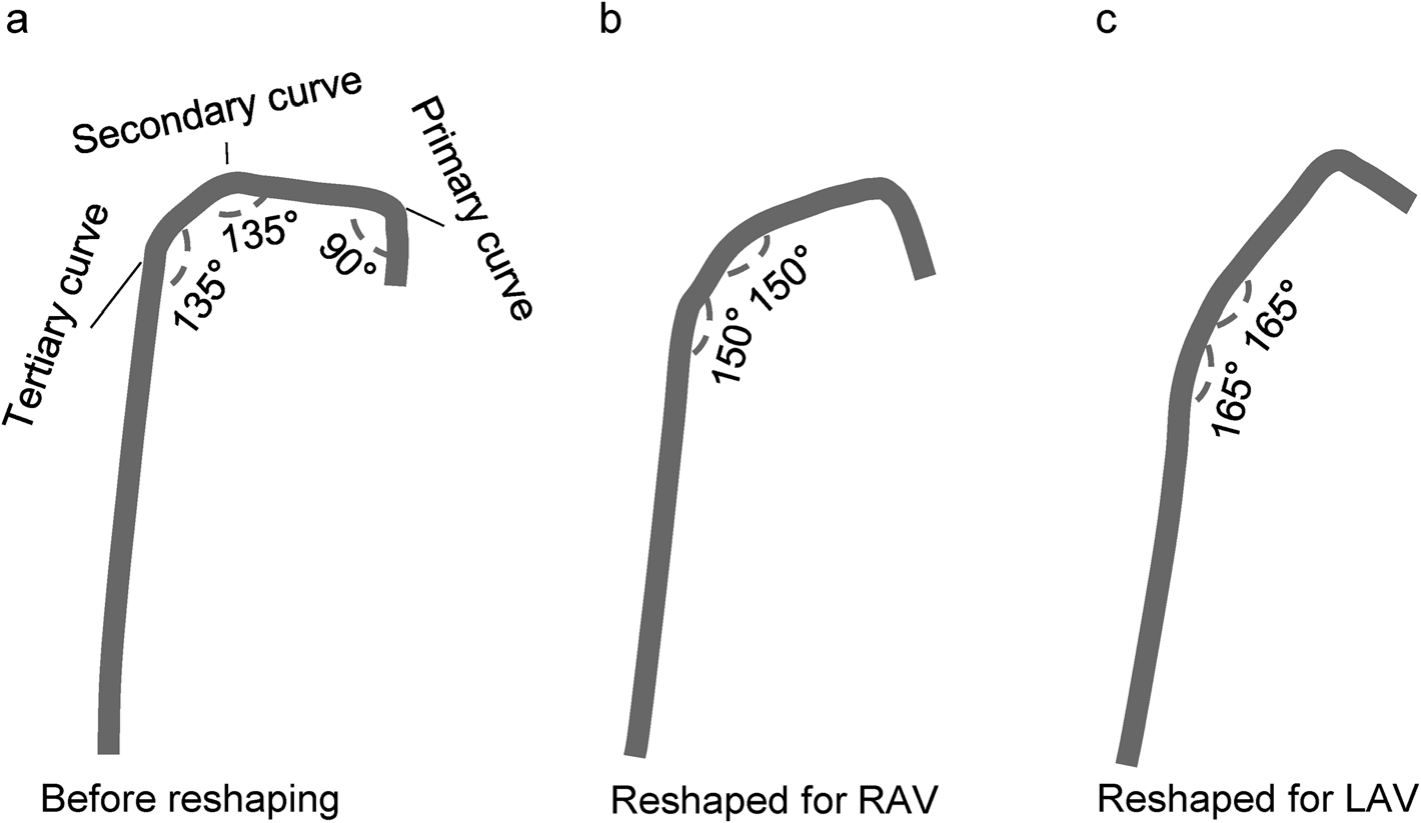
Manual reshaping of Tiger catheters. **a** A 5-Fr Tiger catheter before reshaping. **b** Reshaping of the Tiger catheter for right adrenal vein (RAV) sampling. **c** Reshaping of the Tiger catheter for left adrenal vein (RAV) sampling

**Fig. 2:**
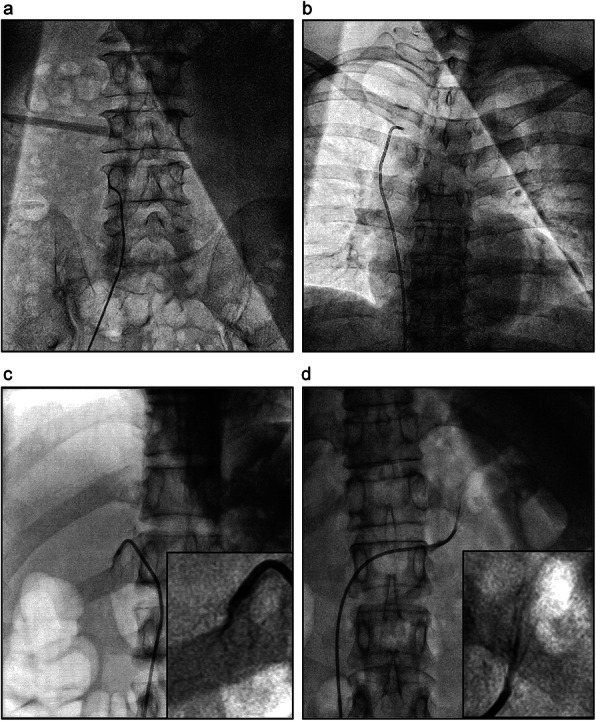
Adrenal vein sampling. Blood sampling from the inferior vena cava (**a**), the superior vena cava (**b**), the right adrenal vein (**c**), and the left adrenal vein (**d**)

Patients were prepared for the AVS procedure according to the guidelines [13]. Briefly, antihypertensive medications were switched to peripheral α1-adrenergic receptor blockers and/or long-acting calcium-channel blockers 4 weeks before the AVS procedure. Hypokalemia, if present, were corrected with potassium supplements before the procedure. The patients were kept in the supine position for 1 h before AVS. AVS was performed in the morning by two inexperienced fellows who were recently enrolled in the interventional cardiology fellowship program. A 5-Fr sheath was percutaneously placed into the right common femoral vein under local anesthesia. A 5-Fr Tiger catheter (Terumo, Tokyo, Japan) was manually reshaped for the right adrenal vein catheterization by slightly stretching the secondary and tertiary curves from 135° to 150° (Fig. 1a and b). The reshaped catheter was inserted into the IVC and then into the superior vena cava using a guidewire (0.035-in. loach guidewire, Terumo Corporation, Tokyo, Japan), and a 5 ml sample of blood was drawn from each site (Fig. 2a and b). The right adrenal vein was catheterized by attempting canalization at around the level of the eleventh thoracic vertebra (Fig. 2c). Catherization into the right adrenal vein was confirmed by angiography (Fig. 2c). After drawing a 5 ml sample of blood from the right adrenal vein, the catheter was withdrawn and manually reshaped again for the left adrenal vein catheterization by further stretching the secondary and tertiary curves from 150° to 165° (Fig. 1c). The reshaped catheter was inserted again into the IVC, and catherization into the left adrenal vein was confirmed by angiography (Fig. 2d). After drawing a 5 ml sample of blood from the right adrenal vein, the catheter was withdrawn. At the end of the procedure, the sheath was removed, and a 10 min manual compression was applied for hemostasis.

### Data collection

The demographic and laboratory data and procedural factors including period of procedure, fluoroscopy time, and procedural success and complication rates were collected from record reviewing (Table 1). The success of AVS was defined as the adrenal/peripheral vein cortisol ratio is more than 2:1 without cosyntropin.

**Table 1:** Patient demographics, laboratory, and procedural data

Items	Data
Age, year	55.4 ± 12.3
Male, n (%)	59 (55.7)
History of hypertension, year	8.5 ± 4.2
Systolic blood pressure, mmHg	147 ± 24
Diastolic blood pressure, mmHg	94 ± 14
Creatinine, μmol/l	68.5 ± 22.3
Plasma potassium, mmol/l	3.4 ± 0.2
Urine potassium, mmol/l	42.5 ± 20.5
Aldosterone renin ratio, pg/ml to ng/(ml·h)	455.9 ± 32.7
Plasma aldosterone before salt loading, ng/l	345.7 ± 132.4
Plasma aldosterone after salt loading, ng/l	192.8 ± 106.5
Success rates of AVS, case (%)	
Left AVS	99 (93.4%)
Right AVS	97 (91.5%)
Bilateral AVS	96 (90.6%)
Procedural time, min	33.0 ± 8.2
Fluoroscopy time, min	5.8 ± 1.7
Radiation dosage, mGy	117.3 ± 25.5
diagnostic contrast, ml	17.3 ± 5.5
Complications, n (%)	
Hematoma at the femoral puncture site	1 (0.9)

*AVS* Adrenal vein sampling

### Statistical analysis

Continuous data are presented as mean ± standard deviation, while categorical data are presented by frequency with percentage or range. A learning curve was drawn based on a cumulative sum (CUSUM) analysis for procedure time. The operation time for each case is defined as *xi*, and the mean procedure time of all the cases is defined as *μ*. Therefore, the CUSUM at procedure time is calculated as $\sum \limits_{i=1}^n\left( xi-u\right)$ [14]. The CUSUM was plotted against the case number.

## Results

### Success rate of AVS procedure

The single Tiger catheter was successfully inserted into the right and left adrenal veins in all 106 patients. The success rate of bilateral AVS was 90.6% (96 out of 106) (Table 1). The procedural and fluoroscopy time was 33.0 ± 8.2 min and 5.8 ± 1.7 min, respectively (Table 1). The radiation exposure for each procedure was 117.3 ± 25.5 mGy, and the contrast agent used per procedure was 17.3 ± 5.5 ml (Table 1). Only 1 patient (0.9%) had a hematoma at the femoral puncture site. No other procedure-related complications were observed.

### Canalization of the right adrenal vein

**Fig. 3:**
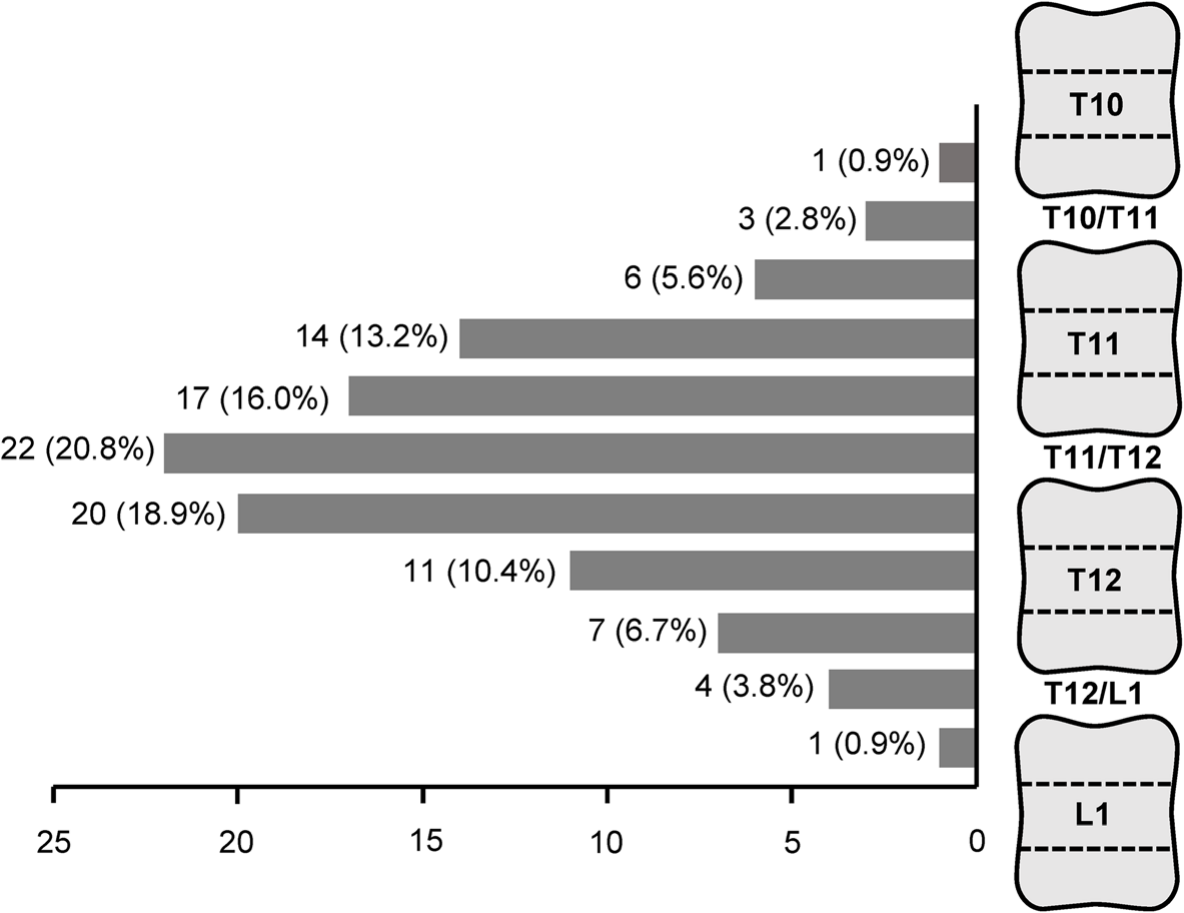
Location of right adrenal vein orifice on the inferior vena cava. Data are cases (%) of right adrenal veins in relation to the levels of vertebral bodies and disks

**Fig. 4:**
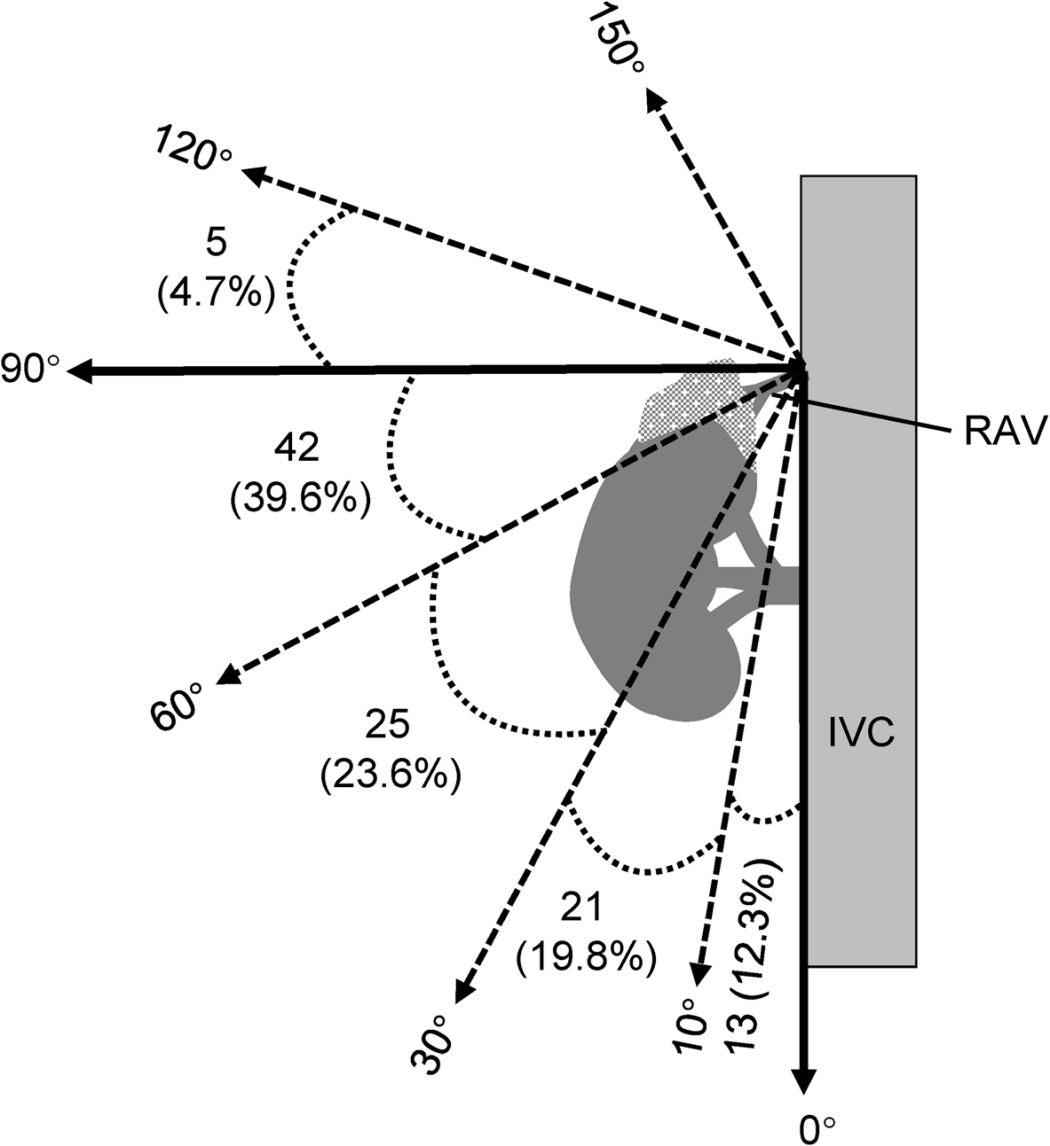
The angle between the right adrenal vein (RAV) and the inferior vena cava (IVC)

Canalization of the right adrenal vein is critical for success of AVS but particularly challenging. The location of the right adrenal vein orifice at the IVC varies from the lower part of the tenth thoracic vertebra and the upper part of the first lumbar vertebra (Fig. 3). Most (79.2%) right adrenal veins drain into the IVC between the middle part of the eleventh thoracic vertebra and the middle part of the twelfth thoracic vertebra (Fig. 3). The angle between the right adrenal vein and the IVC is 57.3 ± 32.5 degree (ranging between 5 to 115 degree) (Fig. 4).

### Learning curve of AVS

**Fig. 5:**
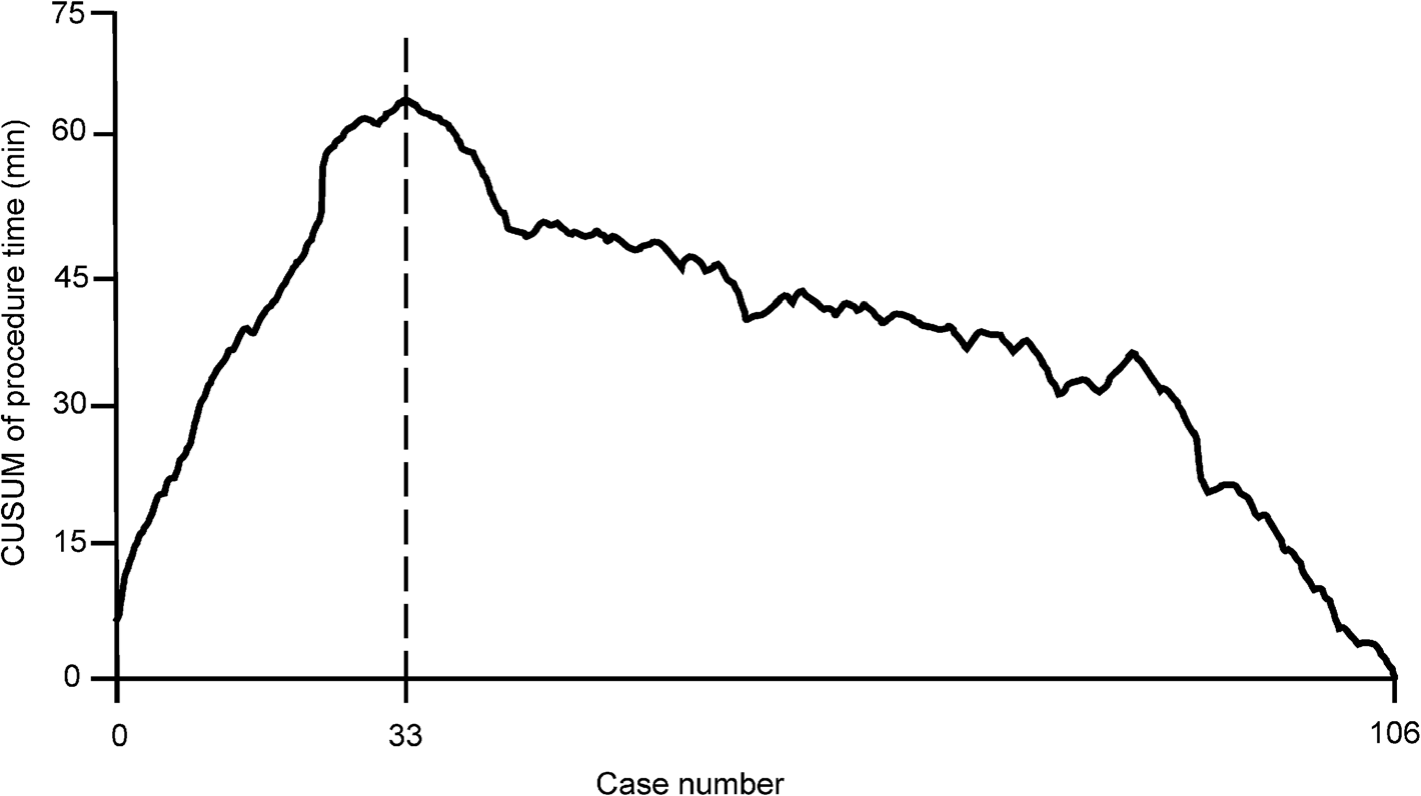
The learning curve of the AVS procedure. The CUMSUM of procedure duration was plotted against case number

The CUMSUM of procedure duration was plotted against case number. Among the 106 consecutive patients, the CUMSUM value inflected at patient number 33 (Fig. 5). Patients 1–32 constituted the learning phase of the curve with procedure duration of 49 ± 14 min, while patients 34–106 consisted of the experienced phase with procedure duration of 30 ± 5 min (Fig. 5).

## Discussion

This study demonstrates that the single-catheter approach is a feasible, effective, and easy-to-learn method for AVS in patients with PA. We found that the single-catheter approach achieved a procedural success rate of 90.6% for bilateral AVS with very minor complications.

AVS has long been considered as a challenging procedure even in experienced hands, and it needs a relatively long learning period [11, 15]. The procedural success rate varies in a broad range and can be as low as around 30% [16, 17]. Although the success rate in the present study is high, we could not make conclusion that the single-catheter approach has higher success rate than other techniques without head-to-head comparisons. The difficulties of the AVS procedure come from the anatomic complexities and variations of the adrenal veins, especially the right adrenal vein. Since the right and left adrenal veins drain into the IVC through distinct courses with different angles, two different catheters are usually used. The Tiger catheter has been used to canalize the left adrenal vein by other groups [18]. The present study demonstrated that a simple manual reshaping could make the Tiger catheter for both left and right adrenal vein catherization. A couple of alternative methods have been developed to improve the right adrenal vein canalization. Specially designed catheters are available for the right adrenal vein sampling [19]. In addition, microcatheters have been used to facilitate the right adrenal vein canalization and to avoid mistakenly sampling from the common trunk of the accessory hepatic and the right adrenal vein [19, 20]. The approaches of using a special catheter or a microcatheter could improve procedural success rate, however, they may increase cost. Technical challenging and expensive materials may impede the wide application of AVS for the subtyping of patients with PA. The single-catheter approach with manual reshaping provides an alternative method to complete AVS.

Catheterization of the right adrenal vein is particularly challenging because it is a relatively small sized vein with anatomical variations. Therefore, localizing the right adrenal vein during AVS is critical for technical success. Matsuura et al. investigated the anatomy of the right adrenal vein using multidetector computed tomography and demonstrated that the orifice of the right adrenal vein was located between the level of the eleventh thoracic and the first lumbar vertebrae, with the majority at a level ranging from the middle third of the twelfth thoracic vertebra to the superior third of the first lumbar vertebra [21]. In the 106 patients enrolled in the preset study, the orifice of most right adrenal veins is slightly higher, i.e. between the middle part of the eleventh thoracic vertebra and the middle part of the twelfth thoracic vertebra. A previous study showed that the anatomical variations of the right adrenal vein based on computed tomography are highly concordant with the findings of angiography [22]. Therefore, computed tomography may provide useful information for localizing the right adrenal vein before AVS. In consistent with the previous study [21], the angle at which the right adrenal vein drains into the IVC varies between 5 to 115 degree in the present observation. As the majority of the right adrenal veins flow into the IVC with an acute angle, some groups suggested performing AVS via the forearm veins as an alternative route to the femoral vein [18].

One of the limitations is that this is a single-arm study. We could not make conclusion regarding whether the single-catheter technique is superior to or easier and cheaper than other AVS approaches. Further head-to-head comparisons are needed to answer those questions in the future. Another limitation is that pharmacological stimulation was not used during sequential AVS. Stress-induced fluctuations in cortisol and aldosterone secretion might affect the lateralization ratio. Although AVS was performed in the morning after the patients kept in the supine position for 1 h to minimize stress, it is better to apply cosyntropin stimulation before sequential AVS.

## Conclusions

In conclusion, the single-catheter approach with repeated manual reshaping provides a feasible and effective method for the AVS procedure.

## Data Availability

Data relevant to this study are available from the corresponding authors upon reasonable request.

## Abbreviations

AVS: Adrenal vein sampling
PA: Primary aldosteronism;
IVC: Inferior vena cava
CUSUM: Cumulative sum
